# Intestinal ZIP8 Regulates Tissue Manganese Distribution and Modifies Manganese Overload in ZIP14 Deficiency

**DOI:** 10.1155/jnme/6717314

**Published:** 2025-12-04

**Authors:** Yuze Wu, Shannon McCabe, Ningning Zhao

**Affiliations:** School of Nutritional Sciences and Wellness, The University of Arizona, Tucson 85721, Arizona, USA

**Keywords:** manganese, metal, nutrient, transport, ZIP14, ZIP8

## Abstract

ZIP8 (SLC39A8) is a metal transporter known to facilitate the uptake of manganese, zinc, and iron, but its role in the intestinal epithelium remains unclear. In this study, we investigated the function of intestinal ZIP8 using intestine-specific *Zip8* knockout (*Zip8*-I-KO) mice and a manganese overload model by crossing *Zip8*-I-KO mice with *Zip*14^−/−^ mice to generate double knockout mice. We confirmed that ZIP8 is localized to the apical membrane of colonic Caco-2 cells, a widely used model for enterocytes. Deletion of intestinal ZIP8 did not affect blood manganese levels under basal conditions but led to significantly reduced manganese concentrations in the liver and bone, suggesting a role in tissue-level manganese distribution. In the ZIP14-deficient background, intestinal ZIP8 deletion resulted in a significant reduction of blood and brain manganese levels in female double knockout mice, while no changes were observed in males. Bone manganese remained elevated in all groups. These findings indicate that intestinal ZIP8 contributes to manganese absorption and distribution with its effects varying depending on sex and may serve as a modifier of manganese overload in ZIP14 deficiency.

## 1. Introduction

ZIP8, also known as SLC39A8 (solute carrier family 39, member 8), is a metal-ion transporter that belongs to the ZIP family, which stands for zinc-regulated transporter (ZRT) and iron-regulated transporter (IRT)–like proteins. ZIP8 is a multi-transmembrane protein capable of transporting various divalent cations, including cadmium, zinc, iron, and manganese [1–4]. Studies of human diseases caused by ZIP8 mutations helped clarify its physiological function. In 2015, two clinical studies linked *ZIP8* mutations to severe manganese deficiency [5, 6]. One study showed that homozygous mutations in *ZIP8* led to severe manganese deficiency [5]. Metal level analysis revealed extremely low to nondetectable blood manganese levels in affected individuals. The clinical phenotypes included intellectual disability, developmental delay, hypotonia, strabismus, cerebellar atrophy, and variable short stature [5]. Another study reported compound heterozygous mutations in *ZIP8* in two patients diagnosed with congenital disorders of glycosylation (CDG) [6]. Manganese was not detectable in blood or urine samples from both patients. Since manganese is essential for glycosyltransferase enzymes, these two patients had defective glycosylation of transferrin, a plasma glycoprotein marker. Like the first report, the clinical phenotypes of both patients included skull deformities, severe seizures, short limbs, psychomotor retardation, and hearing loss [6]. The identification of *ZIP8* mutations and their associated human diseases emphasizes the importance of this metal transporter in maintaining systemic manganese homeostasis.

Systemic manganese homeostasis is primarily regulated by the liver and intestine, which work together to control manganese absorption, distribution, and excretion [7, 8]. ZIP8 is expressed in the liver, where it contributes to manganese retention. In hepatocytes, ZIP8 is localized to the apical membrane, facing the bile canaliculi [9]. This positioning allows ZIP8 to reabsorb manganese from bile, limiting its loss through biliary excretion. Loss of ZIP8 disrupts this process, contributing to systemic manganese deficiency, as observed in individuals with *ZIP8* mutations. However, the liver may not be the only organ responsible for the manganese deficiency seen in ZIP8-related disease. Given the widespread expression of ZIP8 in other tissues [10, 11], it is likely that ZIP8 has additional roles in systemic manganese distribution and cellular uptake that remain to be fully understood.

ZIP8 is also expressed in the intestine [11–13], an organ that plays a central role in manganese absorption and homeostasis. While ZIP8 has been studied in other tissues such as the liver and lung, its specific function in the intestinal epithelium remains poorly understood. This is particularly important because the intestine is the primary site of dietary manganese uptake, and any disruption in transporter function here may directly affect whole-body manganese levels. Although previous studies have identified another ZIP family protein, ZIP14, as a key manganese transporter at the basolateral surface of enterocytes [14], recent findings suggest that ZIP8 is localized to the apical membrane, where it may mediate direct manganese uptake from the intestinal lumen [12]. However, whether intestinal ZIP8 significantly contributes to systemic manganese regulation is still unclear.

To address this knowledge gap, the present study aims to investigate the role of ZIP8 in the intestine using intestine-specific *Zip8* knockout (*Zip8*-I-KO) mice. This model allows for selective deletion of ZIP8 in intestinal epithelial cells, enabling direct assessment of how ZIP8 impacts overall metal homeostasis. Furthermore, this study also investigates whether targeted deletion of intestinal ZIP8 can alleviate manganese loading in a genetic model of manganese overload—*Zip14* knockout (*Zip*14^−/−^) mice. Our results provide critical insights into the physiological function of ZIP8 in the intestine and its contribution to systemic manganese homeostasis.

## 2. Materials and Methods

### 2.1. Animals

All mice were housed in the University of Arizona's central animal facility. The procedures for animal experiments were approved by the Institutional Animal Care and Use Committee. Mice were fed an irradiated NIH-31 diet, which contains about 155 ppm manganese (Teklad 7913; Envigo, Indianapolis, IN), and were housed at 21°C–22°C with a 12 h light/12 h dark cycle. *Villin*-*Cre* and *Ubc*-*Cre*^ERT2^ transgenic mouse lines were obtained from the Jackson Laboratory (Bar Harbor, ME, USA). *Zip*14^−/−^ mice were sourced from the Mutant Mouse Resource & Research Center. *Zip*8^flox/flox^ mice were purchased from Taconic Biosciences (Germantown, NY, USA). Inducible *Zip8*-knockout mice were created in two steps. First, *Zip*8^flox/flox^ mice were crossed with Ubc-*Cre*^ERT2^ mice (Jackson Laboratory, Bar Harbor, ME). The resulting *Zip*8^flox/flox^-*Ubc*-*Cre*^ERT2^ offspring then received tamoxifen by intraperitoneal injection once a day for 5 days. Tamoxifen activated Cre^ERT2^, excised the floxed *Zip8* alleles, and generated *Zip8* inducible knockout (*Zip*8^flox/flox^-iKO) mice. To generate *Zip8*-I-KO mice, *Zip*8^flox/flox^ mice were first mated with *Villin-Cre* transgenics, giving *Zip*8^flox/−^-Villin-*Cre*^+/−^ offspring; these progeny were intercrossed to produce *Zip*8^flox/flox^-*Villin*-Cre^+/−^ mice (*Zip8*-I-KO). To generate *Zip*14^−/−^ mice with *Zip8*-I-KO, *Zip*14^−/−^ mice were bred with *Zip8*-I-KO mice, producing F1 heterozygous offspring. Interbreeding F1 mice generated F2 progeny carrying *Zip*14^−/−^-*Zip*8^flox/flox^-*Villin*-*Cre*^+/−^ alleles; these mice were selected as the double-knockout (DKO) line.

Mouse Direct PCR kit (Bimake, Houston, TX, USA) and the following primers were used to determine mouse genotypes. For *Zip*14^−/−^ mice – DNA506-100: 5′-TCA TGG ACC GCT ATG GAA AG-3′; DNA506-101: 5′-GTG TCC AGC GGT ATC AAC AGA GAG-3′; Neo3a: 5′-GCA GCG CAT CGC CTT CTA TC-3′; DNA506-6: 5′-TGC CTG GCA CAT AGA ATG C-3′. For *Zip*8^flox/flox^ mice – 2476_27: 5′-CAG GGT TTC TCT GTG TAA CAG G-3′; 2474_28: 5′-AGT GTA CAG GCT CCA GCT ACC-3′. For *Ubc-Cre*^ERT2^ mice: 25285: 5′-GAC GTC ACC CGT TCT GTT G-3′; oIMR7338: 5′-CTA GGC CAC AGA ATT GAA AGA TCT-3′; oIMR7339: 5′-GTA GGT GGA AAT TCT AGC ATC ATC C-3′; oIMR9074: 5′-AGG CAA ATT TTG TGT ACG G-3′. For *Villin-Cre* mice: 16775: 5′- GCC TTC TCC TCT AGG CTC GT -3′; 16776: 5′-TAT AGG GCA GAG CTG GAG GA -3′; oIMR9074: 5′-AGG CAA ATT TTG TGT ACG G-3′.

All mice were anesthetized with ketamine/xylazine and sacrificed at ∼9 weeks of age. Blood was collected by cardiac puncture using a syringe and transferred into EDTA-containing tubes and then immediately frozen and stored in liquid nitrogen. After blood collection, tissues were harvested, rinsed briefly in cold 1× phosphate-buffered saline (PBS), dried on a clean paper towel, and stored in liquid nitrogen or at −80°C for later analysis.

### 2.2. Cell Culture and Cell Surface Protein Biotinylation

Caco-2 cells were cultured in DMEM containing 10% fetal bovine serum (FBS) and 1% penicillin–streptomycin. Incubation conditions were 37°C with 5% CO_2_. For the biotinylation study, wild-type Caco-2 cells were seeded on 6-well transwell inserts and grown for 22-23 days. Medium was changed and transepithelial electrical resistance (TEER) was recorded every other day. On the final day of the transwell culture, cells were rinsed twice with 37°C PBS^Ca/Mg^, then twice with ice-cold PBS, and kept on ice. For surface labeling, an ice-cold solution of Sulfo-NHS-SS-Biotin (Thermo Fisher, Waltham, MA, USA) was prepared at 1 mg/mL. For basolateral biotinylation: 1.5 mL Sulfo-NHS-SS-Biotin solution was added to the basolateral chamber and 1 mL PBS to the apical chamber. For apical biotinylation: 1 mL Sulfo-NHS-SS-Biotin solution was added to the apical chamber and 1.5 mL PBS to the basolateral chamber. Plates were rocked gently at 4°C for 30 min. Excess reagent was removed with two cold PBS washes, followed by a 20-minute quench in PBS containing 100 mM glycine and two final cold PBS rinses. Transwell inserts were moved to fresh wells, membranes excised, and cells lysed in 1 mL NETT buffer (150 mM NaCl, 5 mM EDTA, 10 mM Tris-HCl, 1% Triton X-100, pH 7.4) with protease inhibitors. Lysates were vortexed on ice, sonicated (10 × 2 s, 20% amplitude), and cleared (15000*g*, 15 min, 4°C). After saving a 150 μL aliquot for total protein, ∼850 μL was incubated overnight at 4°C with 100 μL NeutrAvidin agarose in spin columns. Beads were washed once with NETT + protease inhibitors, twice with NETT, twice with high-salt wash (350 mM NaCl, 0.1% Triton X-100), and three times with NET buffer. Biotinylated proteins were eluted by soaking beads overnight at 4°C in 100 μL SDS sample buffer (1.7% SDS, 5% glycerol, 150 mM DTT, 58 mM Tris, pH 6.8), then warmed to 37°C for 30 min, and spun down. Eluates and total lysates were frozen at −80°C for later western blotting.

### 2.3. Metal Analysis

For metal measurements, we prepared tissue samples in nitric acid and then sent the digests to the Arizona Laboratory for Emerging Contaminants (ALEC) for inductively coupled plasma mass spectrometry (ICP-MS) analysis. Fresh tissues were weighed in metal-free tubes and dried in an oven until the weight stopped changing. We submerged the dried tissue in 70% nitric acid, let it stand overnight, and then heated it at 80°C for six hours. For blood samples, we added 50 μL of whole blood directly to concentrated nitric acid, left it overnight, and heated it the next day at 80°C for 4 hours. All digests were cooled, spun down to remove debris, and diluted to 3% nitric acid with Milli-*Q* water before analysis.

### 2.4. Western Blotting

Cells and tissue samples were kept on ice and lysed in NETT buffer (150 mM NaCl, 5 mM EDTA, 10 mM Tris-HCl, 1% Triton X-100, pH 7.4) that contained a protease-inhibitor mix. After 10 min of centrifugation at 10000 × *g* and 4°C, the clear supernatant was moved to a fresh tube. Protein levels were quantified with the RC DC™ assay from Bio-Rad. Each sample was then blended with six-fold concentrated Laemmli buffer and warmed at 37°C for thirty minutes to ensure complete denaturation. The prepared proteins were loaded onto a 10% SDS–polyacrylamide gel and separated by electrophoresis. Immediately afterward, the proteins were transferred to a nitrocellulose membrane. To block sites that could bind antibodies nonspecifically, the membrane was soaked for 1 hour in TBST that contained 5% non-fat milk. Next, the blot was placed in primary antibody solution and left to rock overnight at 4°C. We used rabbit antibodies against mouse ZIP8 (1 : 1000), human ZIP8 (1 : 3000), mouse ZIP14 (1 : 1000), and human ZIP14 (1 : 1000). The following morning the membrane was rinsed four times in TBST, 5 min per wash, and then incubated for 1 hr at room temperature with HRP-conjugated goat anti-rabbit IgG diluted at 1 : 3000. After two more washes in TBST and two final washes in TBS, the signals were developed with SuperSignal West Pico chemiluminescent substrate and recorded on a ChemiDoc MP imaging system. To verify equal loading, we stripped the membrane with Restore PLUS buffer for 10 min, blocked it again, and probed with HRP-tagged β-ACTIN or GAPDH antibodies (both 1 : 20,000). When we needed membrane-specific controls, we used mouse antibodies against Na^+^/K^+^-ATPase (1 : 3000) or DMT1 (1 : 5000), followed by the same HRP secondary antibody.

### 2.5. Statistical Analysis

Data analysis was performed using GraphPad Prism (GraphPad Software, San Diego, CA, USA). Unpaired Student's *t*-tests were used to compare means between groups. A *p* value of less than 0.05 was considered statistically significant.

## 3. Results

### 3.1. ZIP8 Is Enriched at the Apical Membrane of Intestinal Epithelial Cells

**Figure 1:**
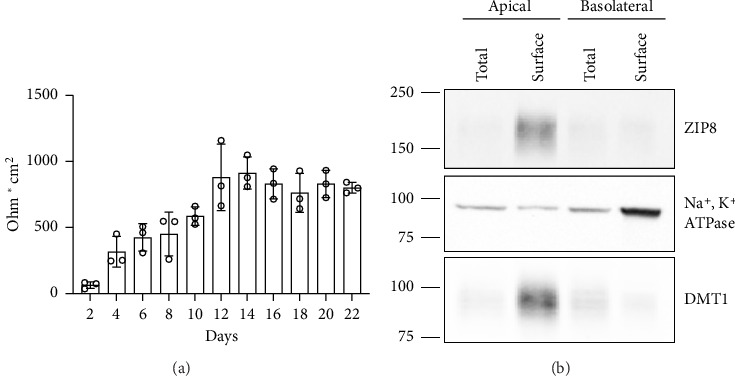
Characterization of Caco-2 transwell culture. (a) Caco-2-WT cells were grown on transwell inserts for 22 days. The transepithelial electrical resistance (TEER) was measured every other day to monitor the formation of a polarized monolayer. The TEER value peaked at around day 12, reaching approximately 800 Ω·cm^2^. This falls within the typical range of 300–1000 Ω·cm^2^, with values above 400 Ω·cm^2^ indicating the formation of a tight monolayer. (b) Basolateral biotinylation and apical biotinylation were performed. Biotin-labeled surface proteins (surface) were isolated from whole-cell lysates (total). Equal amounts of protein were loaded per lane, using Na^+^, K^+^-ATPase as a basolateral marker and DMT1 as an apical marker to confirm successful polarization of the Caco-2 transwell culture. Results represent three independent experiments.

ZIP8 is highly expressed in the distal small intestine and large intestine [12]. To examine its localization in the colon, we used Caco-2 cells grown on transwell inserts, which allow the distinction between apical and basolateral membranes. TEER measurements confirmed the formation of a tight monolayer (Figure 1(a)). We then performed surface biotinylation to detect membrane-localized proteins and to confirm the presence of a polarized monolayer. The results showed that ZIP8 is predominantly expressed at the apical membrane (Figure 1(b) and Figure S1), suggesting a role in mediating luminal manganese uptake in colonic epithelial cells.

### 3.2. Generation and Validation of *Zip8*-I-KO Mice

To test the physiological effect of intestinal ZIP8, we generated *Zip8*-I-KO mice by crossing *Zip*8^flox/flox^ mice with *Villin-Cre* transgenic mice, which drive Cre recombinase expression specifically in intestinal epithelial cells [15]. This breeding strategy allows for the selective deletion of ZIP8 in the intestinal epithelium while preserving ZIP8 expression in other tissues. Littermate *Zip*8^flox/flox^ mice lacking the *Cre* transgene were used as controls.

**Figure 2:**
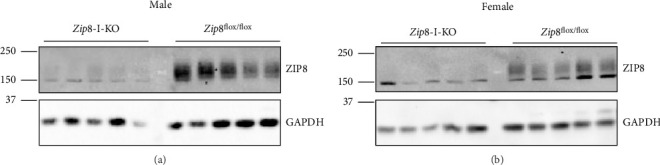
Confirmation of ZIP8 inactivation in the intestine-specific *Zip8* knockout mice. (a) Western blot analysis of ZIP8 protein in large intestine tissue from male control (*Zip*8^flox/flox^) and intestine-specific knockout (*Zip*8^flox/flox^-*Villin-Cre*, *Zip8*-I-KO) mice. (b) The same analysis was performed on female mice. ZIP8 protein is present in control samples but absent in knockout tissues, confirming efficient deletion in the intestinal epithelium. GAPDH was used as the loading control in all blots.

We isolated intestinal tissues from both male and female mice to confirm successful knockout and performed western blot analysis. ZIP8 protein was readily detected in the intestinal tissue of control mice but was not detectable in knockout mice, confirming efficient and specific deletion of ZIP8 in the intestine (Figures 2(a) and 2(b) and Figure S2). This loss of ZIP8 expression was consistent across both sexes and verified the utility of this model for studying the role of ZIP8 in intestinal metal homeostasis.

### 3.3. Blood Levels of Manganese, Iron, and Zinc Remain Unaltered in *Zip8*-I-KO Mice

**Figure 3:**
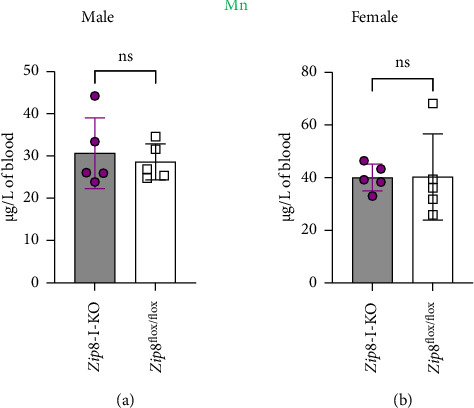
Blood manganese levels are not significantly altered in *Zip8*-I-KO mice. (a) Blood manganese concentrations in male control and *Zip8*-I-KO mice were measured using inductively coupled plasma mass spectrometry (ICP-MS) analysis. (b) Same analysis in female mice. No significant differences were observed between control and knockout groups in either sex (*n* = 5 per group). Data are presented as mean ± standard deviation (S.D.) (ns = not significant).

Blood manganese levels serve as an indicator of systemic manganese status [16]. To determine whether intestinal ZIP8 deletion affects blood manganese, we measured blood manganese concentrations using ICP-MS in both male and female mice (*n* = 5 per group). No significant differences were observed between control and *Zip8*-I-KO mice (Figures 3(a) and 3(b)), indicating that deletion of ZIP8 in the intestine does not significantly impact circulating manganese levels under baseline conditions.

**Figure 4:**
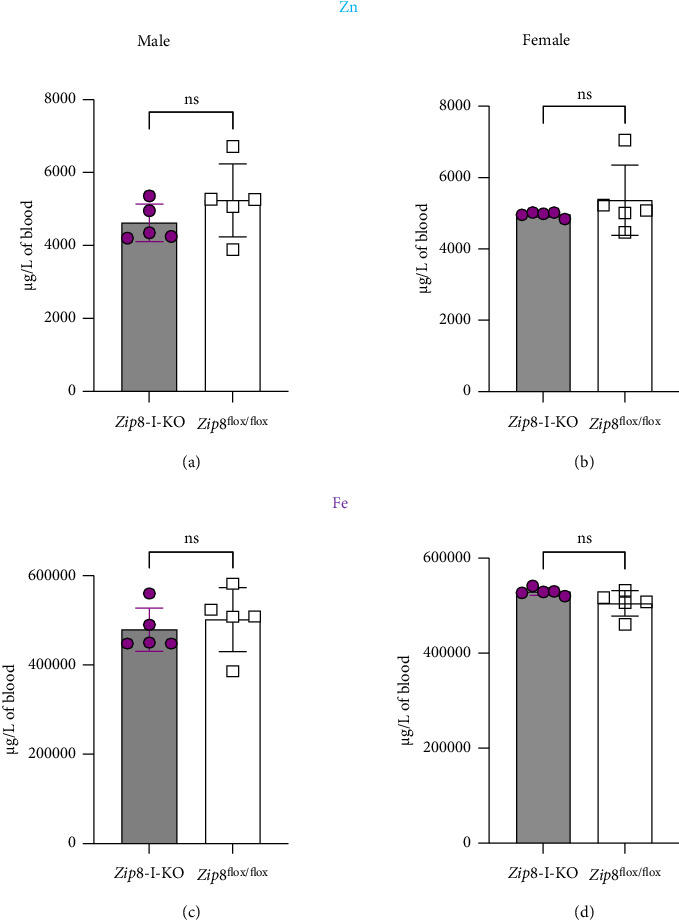
Blood zinc and iron levels are not significantly altered in *Zip8*-I-KO mice. (a, b) Blood zinc concentrations in male and female control and *Zip8*-I-KO mice. (c, d) Blood iron concentrations in male and female control and *Zip8*-I-KO mice. Metal levels were measured by ICP-MS. Data are shown as mean ± S.D. (*n* = 5 per group) (ns = not significant).

Since ZIP8 can transport other essential divalent metals, including zinc and iron, we also measured blood zinc and iron levels in both male and female mice. Using ICP-MS, we found that blood concentrations of zinc (Figures 4(a) and 4(b)) and iron (Figures 4(c) and 4(d)) were not significantly different between *Zip8*-I-KO and control mice. These results suggest that intestinal ZIP8 does not play a major role in regulating systemic levels of zinc or iron under physiological conditions.

### 3.4. *Zip8*-I-KO Mice Have Decreased Manganese in the Liver and Bone

**Figure 5:**
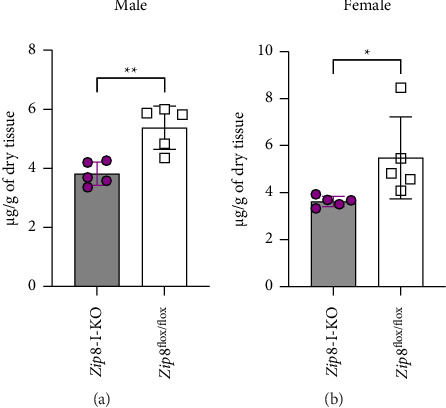
Liver manganese decreases in *Zip8*-I-KO mice. (a) Liver manganese concentrations in male control and *Zip8*-I-KO mice were measured using ICP-MS analysis. (b) Same analysis in female mice. Data are presented as mean ± S.D. (*n* = 5 per group) (^∗^*p* < 0.05, ^∗∗^*p* < 0.01).

**Figure 6:**
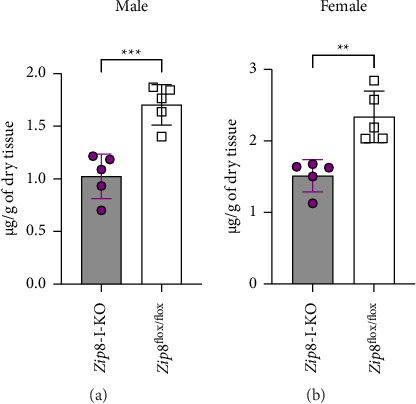
Bone manganese decreases in *Zip8*-I-KO mice. (a) Bone manganese concentrations in male control and *Zip8*-I-KO mice were measured using ICP-MS analysis. (b) Same analysis in female mice. Data are presented as mean ± S.D. (*n* = 5 per group) (^∗∗^*p* < 0.01, ^∗∗∗^*p* < 0.001).

To further investigate whether intestinal ZIP8 deletion affects manganese distribution and storage, we measured manganese concentrations in the liver and bone, two tissues known to store significant amounts of this metal. Using ICP-MS, we analyzed tissue manganese levels in both male and female *Zip8*-I-KO mice and their controls. Our analysis revealed that both male and female *Zip8*-I-KO mice exhibited significantly reduced manganese levels in the liver and bone compared to their wild-type counterparts (Figures 5 and 6). These findings are consistent with a recent study, in which the authors used radiolabeled manganese to demonstrate that intestinal ZIP8 deletion decreases manganese absorption [12]. Together, these results suggest that intestinal ZIP8 acts as an important manganese importer in enterocytes and plays an important role in the maintenance of systemic manganese homeostasis.

**Figure 7:**
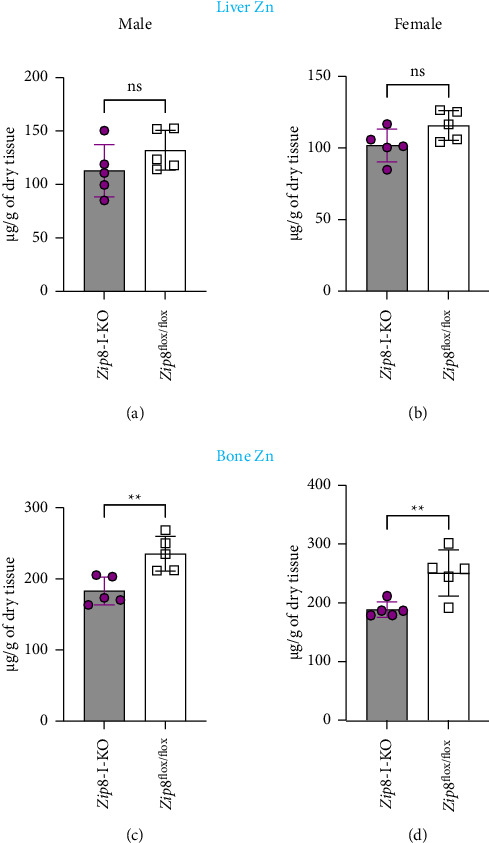
Bone zinc decreases in *Zip8*-I-KO mice. (a) Liver zinc concentrations in male control and *Zip8*-I-KO mice were measured using ICP-MS analysis. (b) Liver zinc analysis in female mice. No significant differences were observed in liver zinc levels between control and knockout groups in either sex (*n* = 5 per group). (c) Bone zinc concentrations in male control and *Zip8*-I-KO mice were measured using ICP-MS analysis. (d) Same analysis in female bone samples. Data are presented as mean ± S.D. (*n* = 5 per group) (^∗∗^*p* < 0.01).

Although the focus of this study was manganese, zinc was included in the same standard set as manganese in the ICP-MS analysis; therefore, we also obtained data on zinc levels in the liver and bone. Our analysis showed no significant difference in liver zinc concentrations between wild-type and *Zip8*-I-KO mice in both male and female groups (Figures 7(a) and 7(b)). Interestingly, both male and female *Zip8*-I-KO mice showed significantly lower bone zinc levels compared to their sex-matched wild-type counterparts (Figures 7(c) and 7(d)). These findings suggest that intestinal ZIP8 may also influence zinc homeostasis; however, the underlying mechanisms remain unclear and require further investigation in future studies.

### 3.5. Deletion of ZIP8 in the Intestine Ameliorates Manganese Loading in Female *Zip*14^−/−^ Mice

**Figure 8:**
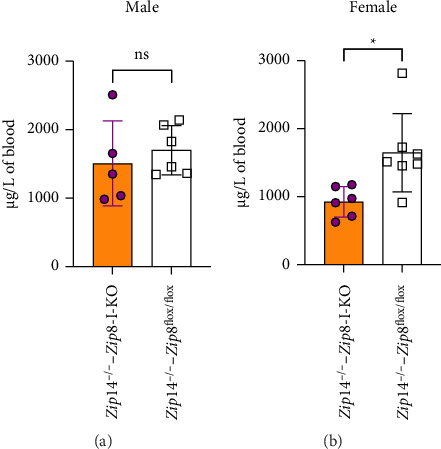
Blood manganese levels are reduced in female but not male double knockout (DKO) mice. (a) Blood manganese concentrations in male control and DKO (*Zip*14^−/−^-*Zip8*-I-KO) mice. (b) Blood manganese concentrations in female control and DKO mice. Manganese levels were measured using ICP-MS. A significant reduction in blood manganese was observed in female DKO mice, while no significant difference was found in males (*n* = 5 to 6 per group). Data are shown as mean ± S.D. (ns = not significant; ^∗^*p* < 0.05).

In an attempt to further investigate the role of ZIP8 in manganese homeostasis, we generated a DKO mouse model by crossing *Zip8*-I-KO mice with *Zip*14^−/−^ mice, a well-characterized genetic manganese overload animal model [17–19]. Loss of ZIP14 function has been previously reported to result in manganese overload in the brain and blood in mice [19, 20], making this model suitable for evaluating whether ZIP8 deletion in the intestine can influence manganese accumulation in a genetic manganese overload background. We compared blood manganese levels between *Zip*14^−/−^ and DKO mice to assess the effect of intestinal ZIP8 deletion in a ZIP14-deficient background. ICP-MS analysis revealed that female DKO mice had significantly reduced blood manganese levels compared to controls (by about 43%), whereas no significant difference was observed in male mice (Figure 8). These results suggest a sex-specific effect of intestinal ZIP8 deletion on systemic manganese levels in the context of ZIP14 deficiency.

**Figure 9:**
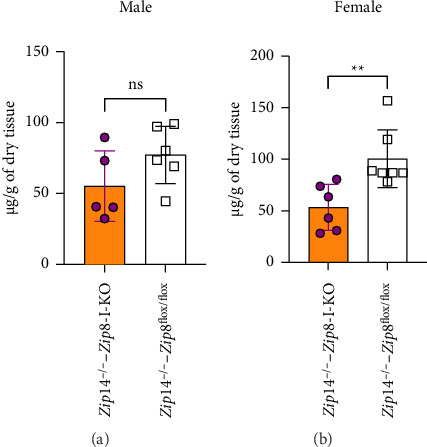
Brain manganese levels in DKO mice. (a) Brain manganese concentrations in male control and DKO mice. (b) Brain manganese concentrations in female control and DKO mice. Manganese levels were measured using ICP-MS. Female DKO mice showed a ∼50% reduction in brain manganese, while male DKO mice exhibited a nonsignificant trend toward reduction (*n* = 5 to 6 per group). Data are shown as mean ± S.D. (ns = not significant; ^∗∗^*p* < 0.01).

**Figure 10:**
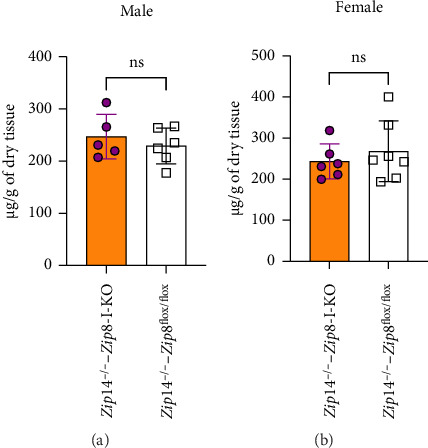
Bone manganese levels in DKO mice. (a) Bone manganese concentrations in male control and DKO mice. (b) Bone manganese concentrations in female control and DKO mice. Manganese levels were measured by ICP-MS. No significant differences were observed between DKO and control mice in either sex (*n* = 5 to 6 per group). Data are presented as mean ± S.D. (ns = not significant).

Previous studies have shown that in *Zip*14^−/−^ mice aged 6–12 weeks, brain manganese levels are approximately 10-fold higher and bone manganese levels are more than 30-fold higher compared to wild-type controls [18]. To determine whether intestinal ZIP8 deletion can reduce manganese accumulation in these organs, we measured brain and bone manganese concentrations in *Zip*14^−/−^ mice and DKO mice. Our results showed that in female DKO mice, brain manganese levels were reduced by approximately 47% compared to *Zip*14^−/−^ controls. In male mice, there was a trend toward decreased brain manganese, but the difference did not reach statistical significance (Figure 9). In contrast, bone manganese levels remained elevated, and no significant reduction was observed in either male or female DKO mice (Figure 10). These findings suggest that intestinal ZIP8 deletion partially alleviates manganese accumulation in the brain, particularly in females, but does not affect bone manganese burden in the ZIP14-deficient background.

## 4. Discussion

This study aimed to investigate the role of ZIP8 in the intestine and its contribution to manganese regulation in vivo. While ZIP8 has been identified as a transporter of multiple essential divalent metals, including manganese [5, 6], its function in intestinal epithelial cells remains poorly understood. Using a tissue-specific knockout approach, we show that deletion of ZIP8 in the intestinal epithelium leads to changes in tissue manganese distribution, particularly in the liver and bone, but does not significantly affect blood manganese levels under basal conditions.

We confirmed that ZIP8 is expressed at the apical membrane of Caco-2 cells cultured on transwells, which is a widely used epithelial model that mimics enterocytes. This localization of ZIP8 supports its potential role in direct manganese uptake from the intestinal lumen. In *Zip8*-I-KO mice, we observed a significant reduction in manganese levels in both liver and bone tissues. These findings suggest that ZIP8 contributes to intestinal manganese absorption, which in turn influences manganese distribution to peripheral tissues. However, the absence of a detectable change in blood manganese concentrations indicates that intestinal ZIP8 is not a major determinant for circulating manganese levels, likely because the body has an intricate system to tightly regulate blood manganese concentrations. This idea is supported by our observation that liver and bone manganese levels were reduced in *Zip8*-I-KO mice. Both the liver and bone serve as storage sites for manganese; it is plausible that intestinal ZIP8 deletion reduces manganese absorption, which could otherwise lead to a drop in circulating levels. However, to compensate for the loss of intestinal ZIP8, the body may adjust manganese distribution by mobilizing less manganese into storage tissues such as the liver and bone or by releasing more manganese from these storage sites into the circulation, thereby preserving manganese concentrations in the blood.

To assess the function of intestinal ZIP8 in a manganese overload setting, we generated a DKO model by crossing *Zip8*-I-KO mice with *Zip*14^−/−^ mice. ZIP14 deficiency is known to cause manganese accumulation in the blood and brain due to impaired hepatic clearance and intestinal retention [14, 17, 18, 21–23]. Deletion of intestinal ZIP8 resulted in a significant reduction of blood manganese in female DKO mice, but not in males, suggesting a sex-dependent effect. Importantly, brain manganese levels were reduced by approximately 47% in female DKO mice, indicating that intestinal ZIP8 contributes to manganese accumulation in the brain under conditions of ZIP14 loss. While a similar trend was observed in males, it did not reach statistical significance. These results indicate that when ZIP14 is inactivated, manganese absorption from the intestine increases while biliary excretion decreases, leading to a drastic rise in total body manganese burden. Under these conditions, the body's normal regulatory system for maintaining manganese balance becomes disrupted. In this altered state, deleting intestinal ZIP8 can reduce manganese absorption from the gut. As a result, blood and brain manganese levels are lowered, but this effect was observed only in female mice. These findings suggest that the role of intestinal ZIP8 in regulating manganese becomes more apparent under overload conditions and may be influenced by sex-specific factors. In contrast, bone manganese levels remained elevated in DKO mice, with no significant reduction observed in either sex, suggesting that manganese accumulation in bone may be less sensitive to changes in intestinal uptake or may be governed by additional regulatory mechanisms.

By examining both basal and ZIP14-deficient conditions, our study shows a functional role for intestinal ZIP8 in manganese absorption and tissue-level distribution, particularly in the condition of impaired systemic manganese clearance. Our analysis of tissue manganese distribution in the *Zip*14^−/−^ mice with intestinal ZIP8 deletion highlights that ZIP8 deletion, while alleviating manganese overload, does not fully normalize manganese levels to those of wild-type mice. This may be due to the loss of hepatic ZIP14 in *Zip*14^−/−^ mice, which impairs hepatic manganese uptake required for biliary excretion [17, 24]. Importantly, this work represents the first in vivo study to evaluate the efficacy of targeting intestinal ZIP8 as a potential strategy to alleviate manganese loading in a genetic model of manganese dysregulation.

Our findings also suggest a potential sex-dependent regulation of manganese homeostasis when ZIP8 is inactivated in the intestine, but the underlying mechanisms remain unclear. The sex differences in manganese metabolism have been well documented in humans, with women generally exhibiting higher blood manganese concentrations than men [25–27]. The underlying mechanism may involve regulation by sex hormones. Our previous mouse study also reported sex-dependent regulation of manganese transporters [28]. Future studies incorporating hormonal modulation, analysis of transporter expression in both sexes, and measurement of other metal levels will help clarify how these factors interact to maintain systemic manganese homeostasis and broader metal metabolism.

## Data Availability

The datasets analyzed in this study are available from the corresponding author upon request.

## Supporting Information

Figures S1 to S2: uncropped original western blot images.
