# Supplementary material for: Intestinal ZIP8 Regulates Tissue Manganese Distribution and Modifies Manganese Overload in ZIP14 Deficiency
Source: J Nutr Metab. 2025 Dec 4;2025:6717314. doi: 10.1155/jnme/6717314 (PMC12698256; doi:10.1155/jnme/6717314)
Supplement: Supporting Information — Additional supporting information can be found online in the Supporting Information section. [file 6717314.f1.pdf]

Intestinal ZIP8 regulates tissue manganese distribution and modifies manganese overload in ZIP14 deficiency

Yuze Wu, Shannon McCabe, and Ningning Zhao*

School of Nutritional Sciences and Wellness, The University of Arizona, Tucson, AZ 85721, U.S.A.;

***Correspondence: zhaonn@arizona.edu; Tel.: (1-520-621-9744)

Supplemental Figures S1 and S2
(Uncropped Western Blot Images for Figures 1B and 2)

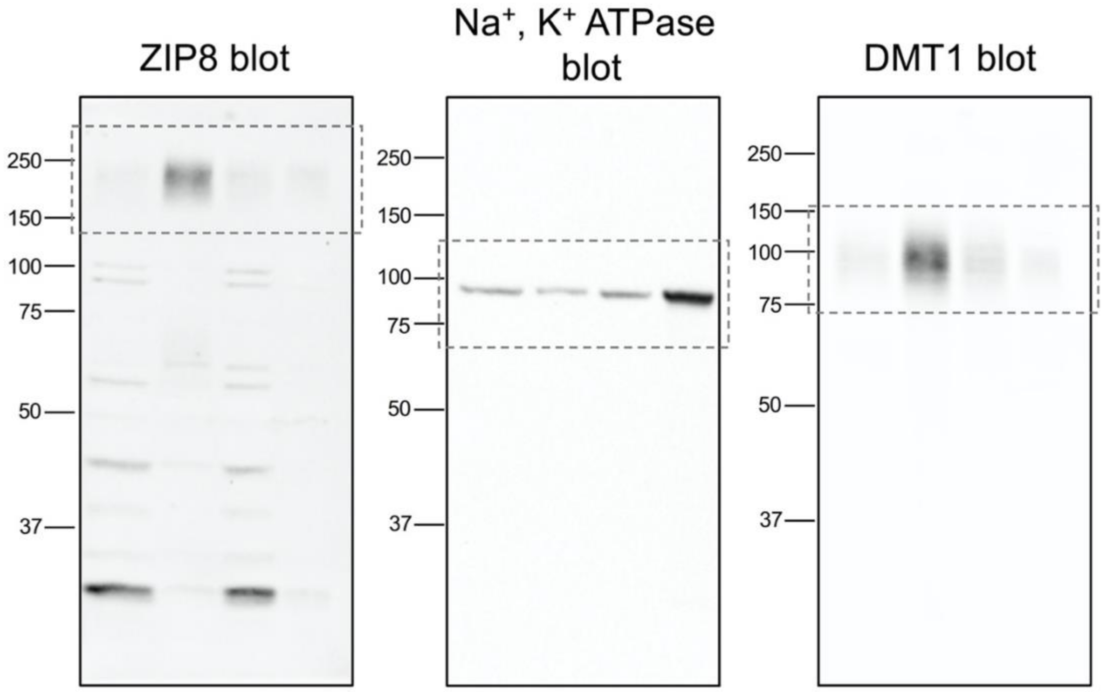

Figure S1. Uncropped Western Blot Images for Figure 1B.

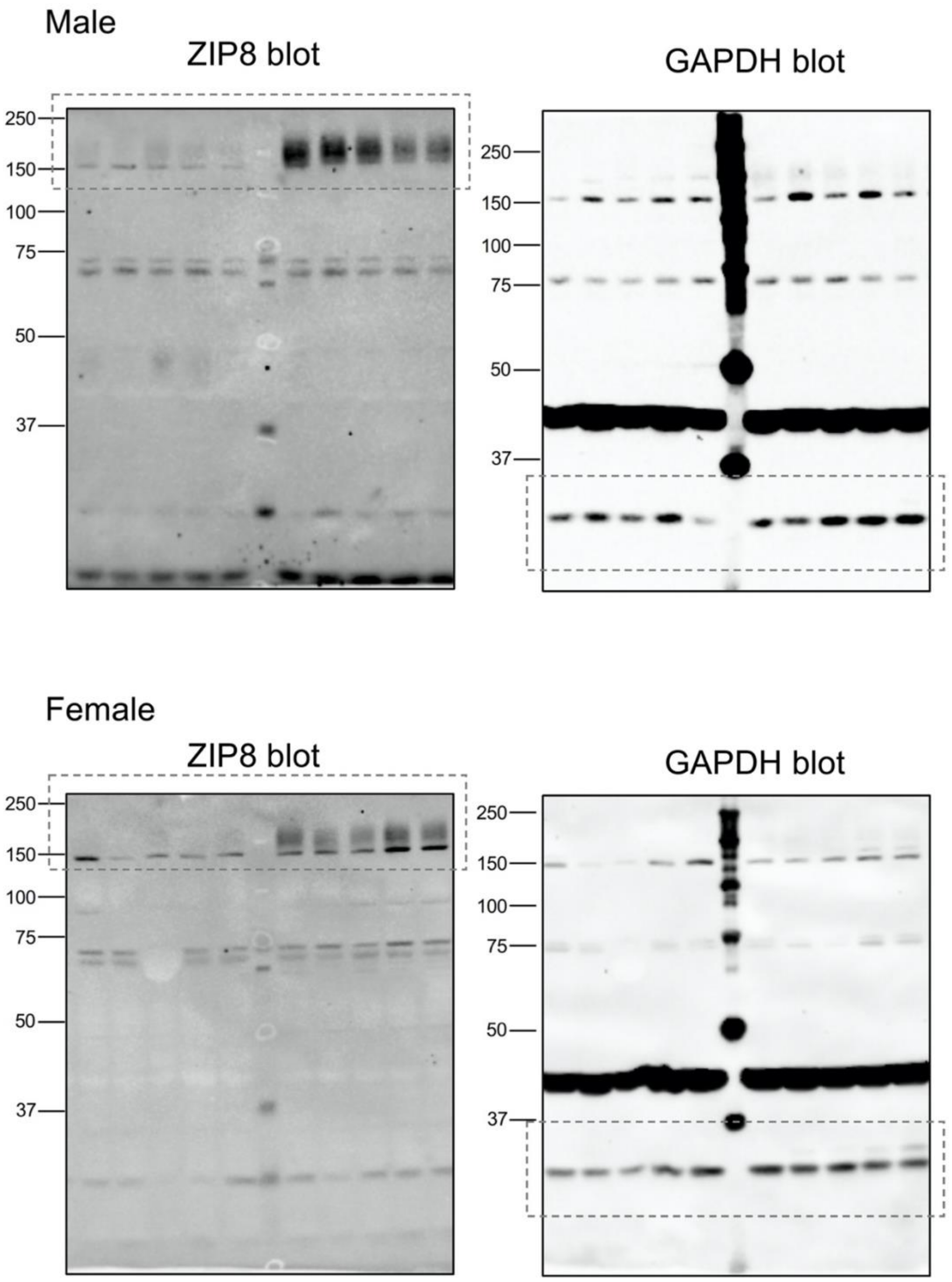

Figure S2. Uncropped Western Blot Images for Figure 2.
